# Comparative study of the effects of submucosal cauterization of the inferior turbinate with or without outfracture

**DOI:** 10.1016/S1808-8694(15)30039-2

**Published:** 2015-10-19

**Authors:** Antonio Celso Nunes Nassif Filho, Carlos Roberto Ballin, Carlos Augusto Seiji Maeda, Gustavo Fabiano Nogueira, Matheus Moschetta, Danielle Salvatti de Campos

**Affiliations:** a Head of the Otolaryngology Department of the Santa Casa de Misericórdia, Curitiba - Brazil.; b Otolaryngologist and Craniofacial surgeon of the Department of Otolaryngology of the Santa Casa de Misericórdia. Curitiba - Brazil.; c 2nd year otolaryngology resident of the Santa Casa de Misericórdia, Curitiba - Brazil.; d 2nd year otolaryngology resident of the Santa Casa de Misericórdia, Curitiba - Brazil.; e 3rd year otolaryngology resident of the Santa Casa de Misericórdia, Curitiba - Brazil.; f 1st year otolaryngology resident of the Santa Casa de Misericórdia, Curitiba - Brazil.

**Keywords:** submucosal cauterization, inferior turbinate hypertrophy, turbinate outfracture

## Abstract

**Aim:**

The objective of this study was to compare the effects of submucosal cauterization of the inferior turbinate with or without outfracture.

**Study Design:**

clinical prospective.

**Method:**

Twenty patients with inferior turbinate hypertrophy were randomized and divided into two groups. The first one was submitted to submucosal cauterization associated with outfracture, and the second one without fracture. Five items were assessed to compare both methods: pain, nasal bleeding, scarring - analyzed through anterior rhinoscopy, observing edema, hyperemia and seropurulent secretion; crust formation (seen through anterior rhinoscopy); and nasal airway patency. Follow-up was performed on days 7, 14, 30.

**Results:**

In both groups crusting formation was similar. Scarring showed better results in the outfracture group in the first two weeks postoperative. The analysis of nasal airway patency showed good results in 80% of the patients submitted to submucosal cauterization with outfratcture on day 30 postoperatively.

**Conclusions:**

We concluded that submucosal cauterization of inferior turbinate with outfracture is better than the procedure without outfracture.

## INTRODUCTION

Nasal turbinates are arch-like bony structures that lay antero-posteriorly in the nasal cavities, having a border attached to the lateral nasal wall and a free medial ledge. There may be three or four nasal turbinates and, despite presenting similar framework, they bear distinct origins. The inferior turbinate, being larger and the one more easily seen, has its own bone, called inferior nasal conchae, anchored to the maxilla, lacrimal, ethmoid and palatine bones. This turbinate tail has a posterior boneless protuberance that bulges into the choana, and it is formed almost exclusively by vascular erectile tissue, which is usually hypertrophic in nasal diseases. The middle, superior and supreme turbinates (the latter not always present), bear a bony structure formed by projections of the ethmoid bone. There is a modest build-up of vascular erectile tissue on the middle turbinate tail, lesser however than that of the inferior turbinate.

The nasal lateral wall, formed by the turbinates and the meatus, has a rather important role in nasal physiology as far as balancing temperature, moisture and also filtering of suspended particles present in inhaled air are concerned. Diseases that cause chronic nasal obstruction – being alergic rhinitis the most prevalent – basically involve the lateral wall of the nasal cavity, causing changes to both the mucosa and the submucosa of the nasal turbinates.

Most rhinopathies cause nasal obstruction, of which the initial treatment is usually clinical. The initial control of environmental factors maybe followed by anti-histamines, topic or systemic steroids and immunotherapy. Surgery is indicated for non-responders. The reduction of nasal turbinates is a surgical approach that has been well discussed in the scientific literature, bearing varied indications and techniques. Among described approaches are the injection of sclerogenic substances and corticosteroids, cryosurgery, total resection of the inferior turbinate, laser ablation surgery, lateral fracture of the turbinate and submucosal ablation by electrocautery. The latter was initially described by Neres in 1907 and by Horn in 1908. In 1930, Beck reported the use of unipolar cautery; and in 1931, Hurd used bipolar ablation for the first time, a technique still broadly used^2^. In 1989, Woodhead et al. reported on the changes to the inferior turbinate caused by submucosal electrocautery ablation. It introduces mucosal changes that lead to fibrosis, apart from reducing the turbinate enlargement potential. The advantage of such procedure is that it can be performed under local anesthesia and it is technically simple to be performed. The major disadvantage is the short duration of the results, ranging from months to years. Meredith et al. reported that 31% of the ablated patients had relapsing symptoms within 33 months3. Differently from turbinectomy – which is irreversible – this procedure can be performed numerous times. Electrocautery ablation usually causes edema and crust formation within 3 to 6 weeks after surgery1. About 20 to 30% of the cases may present adherence. Besides, bone necrosis of the inferior conchae may also occur if the bone is ablated during the procedure, which may then produce bone sequester, edema and erythema of long stand. Electrocautery ablation usually produces good postoperative hemostasis; notwithstanding, 6% of patients may present with late epistaxis^3^. The procedure should be very carefully performed so as to avoid burning of the nasal ala, the columellae, the nasal septum and other structures.

Submucosal ablation is usually performed together with the lateral fracture of the inferior turbinate. However, the turbinate tends to return to its original position after an undetermined amount of time^3^. Thus, the real need for using the lateral fracture coupled with different surgical techniques of the inferior turbinate is yet to be ascertained. From these grounds, the author decided to make a clinical assessment of the patients who underwent submucosal electrocautery ablation with and without lateral fracture of the inferior turbinate, in the treatment of chronic hypertrophic rhinitis.

## OBJECTIVES

Comparative analysis of patients submitted to submucosal electrocautery ablation with and without lateral fracture of the inferior turbinate.

## MATERIAL AND METHODS

We carried out a clinical prospective trial of patients bearing surgical indication of electrocautery ablation of the inferior turbinates to treat nasal obstruction observed in the otolaryngology outpatient ward of the Santa Casa de Misericórdia Hospital in Curitiba, Brazil. The goal of the study was to compare electrocautery ablation performed with and without lateral fracture of the turbinate. The trial comprised 20 patients between 13 and 45 years of age who underwent the surgical procedure in the aforementioned hospital, between October and December of 2003. All the patients underwent a medical interview and complete otolaryngology/head and neck physical exam. Both rigid and flexible endoscopes were used to assess the nasal cavities and the nasopharynx. The criteria used for surgical indication were: bilateral chronic nasal obstruction, unresponsiveness to clinical treatment, no previous nasal surgery, and disease-free nasopharynx^4^. The exclusion criteria were: nasal septum deviation, nasal polyps, and hypertrophic adenoids. The trial included both genders without any specific selection criterion. The patients were randomly assigned to two groups of 10 people each. Group 1 patients underwent submucosal electrocautery ablation of the inferior turbinate coupled with lateral fracture, and group 2 underwent submucosal electrocautery ablation alone, without lateral fracture. The patients were assessed on the 1st, 7th, 14th and 30th day of post-op according to the protocol (attached). Breathing was assessed through the Likert Visual Analog Scale (0 to 10).

All patients underwent the following surgical procedure:

1. Topic anesthesia with 10% xylocaine spray;
2. Sedation with midazolam hydrochloride and fentanyl citrate, or general anesthesia, depending on the case;
3. Local anesthesia with 2% xylocaine and epinephrine, without vasoconstrictor, in a 1:100,000 concentration.
4. Infiltration of the previous solution along the inferior turbinate;
5. Submucosal electrocautery ablation along the head, tail and body of the inferior turbinate by Wavetronic bipolar electrocautery (groups 1 and 2);
6. Medial fracture of the inferior turbinate (group 1);
7. Lateral refracture of the turbinate (group 1);
8. Anterior nasal packing with terramycin ointment and bismuth subgallate;
9. Identical procedure performed on the contralateral turbinate.

## RESULTS

### Demographics

The demographics of the trial groups did not show any significant difference as far as age and gender were concerned; in other words, age and gender distribution was practically the same in the assessed groups. Their ages ranged between 13 and 45 as shown in Table 1.

**Table 1:** Demographics of the trial groups.

Assessment	with (n=10)		without (n=10)		total (n=20)		p value
	N	%	Nº	%	1º	%	
Age (years)							0,733(1)
Average ± standard deviation	22,0 ± 10,2		21,9 ± 7,9		22,0 ± 8,9		
Minimum and maximum	13,0 e 45,0		13,0 e 32,0		13,0 e 45,0		
Median	45,0		32,0				

Gender							0,655(2)

Male	04	40,0	06	60,0	10	50,0	
Female	06	60,0	04	40,0	10	50,0	

(1) Mann-Whitney.

(1) Chi-squared.

### Day 1 post-op assessment

On the 1^st^ day of post-op assessment, the groups did not show significant difference as far as nasal bleeding and pain were concerned. Regardless of the surgical technique (with or without lateral fracture), none of the patients presented nasal bleeding that would require nasal packing. Moreover, most of the patients did not report pain that called for potent analgesia (opioid derivatives) (Table 2).

**Table 2:** Day 1 post-op assessment of the trial groups.

Assessment	with (n=10)		without (n=10)		Total (n=20)		p value(1)
	No	%	N°	%	N°	%	
Nasal Bleeding							0,500
None	06	60,0	07	70,0	13	65,0	
Mild	04	40,0	01	10,0	05	25,0	
Moderate	-	-	02	20,0	02	10,0	

Pain							0,237

None	-	-	02	20,0	02	10,0	
Mild	08	80,0	07	70,0	15	75,0	
Moderate	02	20,0	01	10,0	03	15,0	

(1) Fisher's Exact.

### Day 7 post-op assessment

On the 7^th^ day post-op assessment (Table 3), the trial groups did not show any significant difference in terms of crust and nasal permeability. As for healing, the fracture group presented good healing (60.0%), while the group without fracture presented moderate healing (70.0%) (p=0.041) (Figure 1).

**Table 3:** Day 7 post-op assessment of the trial groups.

Assessment	with (n=10)		without (n=10)		Total (n=20)		p value(1)
	N°	%	N°	%	N°	%	
Healing							0,041
Good	06	60,0	01	10,0	07	35,0	
Moderate	04	40,0	07	70,0	11	55,0	
Poor	-	-	02	20,0	02	10,0	

Crusts							0,325(2)

None	05	50,0	07	70,0	12	60,0	
Moderate	05	50,0	03	30,0	08	40,0	

Nasal permeability							0,171

Poor	-	-	03	30,0	03	15,0	
Moderate	07	70,0	05	50,0	12	60,0	
Good	03	30,0	02	20,0	05	25,0	

(1) Chi-squared.

(2) Fisher's Exact.

**Chart 1:**
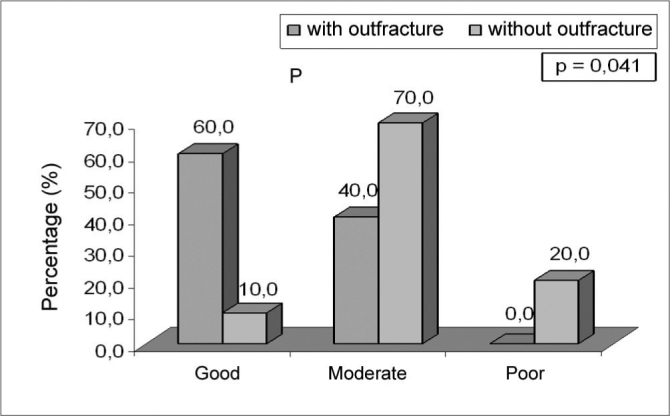
Healing after 7 days of surgery of the trial groups.

**Chart 2:**
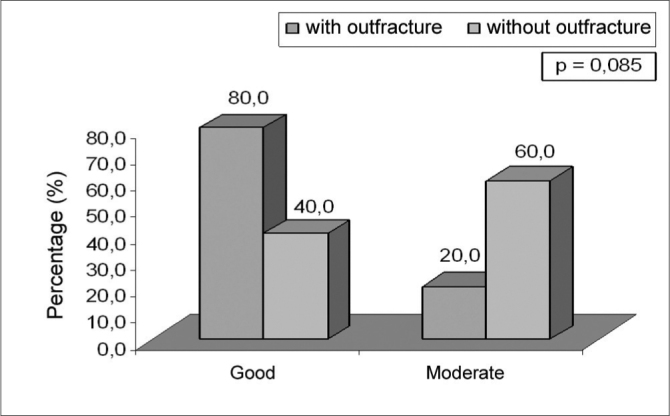
Healing after 14 days post-op of the trial groups.

**Chart 3:**
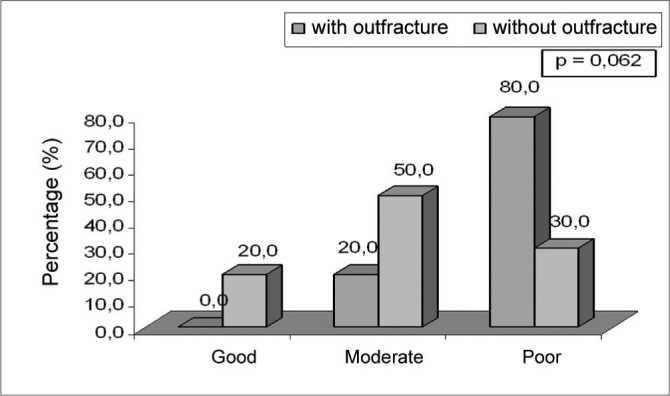
Nasal permeability after 30 days post-op of the trial groups.

### Day 14 post-op assessment

On the 14^th^ day of post-op assessment, the trial groups did not show significant differences as far as crusts and nasal permeability are concerned. As for healing, no significant difference was seen. However, we must highlight the fact that the lateral fracture group showed good healing (80.0%) while the group without fracture presented moderate healing (60.0%) (p=0.085) (threshold likelihood indicating a trend) (Figure 2).

### Day 30 post-op assessment

On the 30^th^ day post-op assessment, no significant difference was seen on the trial groups as far as healing and crusts were concerned. Regarding nasal permeability, no significant difference was seen. However we must highlight the fact that the group with lateral fracture presented good nasal permeability (80.0%) while the non-fractured group had moderate permeability (50.0%) (p=0.062) (threshold likelihood indicating a trend) (Figure 3).

There were very few complications seen in the present study. Only one patient after 30 days developed posterior nasal synechiae in the right nasal cavity, which meant 5% of the cases considered. No patient complained of intense pain or late epistaxis on the follow-up. We used descriptive data analysis through tables, charts and figures. Non-parametric tests - “Mann-Whitney” (through the “Primer of Biostatistics” software), “Chi-squared” and “Fisher's Exact” (through the “Epi-Info” software) were utilized in order to corroborate the goal stated in this paper. The significance level (significance probability) used was lower than 5% (p<0.05).

## DISCUSSION

There are a number of surgical methods described in the literature for the treatment of chronic hypertrophy of the inferior turbinate. However, most of the authors concur that the initial clinical treatment must be thoroughly attempted before patients are referred to surgery. Each approach has its effectiveness, duration and potential complications, all of which must be taken into consideration at first hand. The choice of technique to be used varies according to the surgeon's training and his/her familiarity with the method. There is no consensus in the literature as to which technique is more effective for the surgery of the inferior hypertrophic turbinate. Notwithstanding, the authors do agree that the treatment should be the one that brings about the least morbidity to the patients. Among the many methods presented in the literature, the submucosal electrocautery ablation is one of the most widely used.

The literature shows distinct opinions about the inferior turbinate submucosal electrocautery ablation. To begin with, the amount of coagulated tissue is difficult to be measured. According to Wengraf et al. (1986), there are no precise parameters to be used in the calculation of the amount of tissue to be ablated^5^. Likewise, in our trial we noticed that the amount ablated varied according to our experience. However, Williams et al. state that the most common complications are late hemorrhage, crust formation and long standing rhinorrhea^6^. Contrary to this previous author, we did not observe late hemorrhage nor complaints of prolonged rhinorrhea, or any account of intense pain in the follow-up of our patients. Crust formation, however, was observed as early as in the first week. Nonetheless, we did not observe intense crust formation in any patient. Jackson et al. described the onset of nasal synechiae after submucosal electrocautery ablation in 20 to 30% of the cases1. In our study, only one patient developed nasal synechiae after surgery, and he belonged to the group of patients who underwent electrocautery ablation without lateral fracture. Despite such disadvantages, submucosal electrocautery ablation remains the procedure of choice for a large number of otolaryngologists, as it is a technically simple method to be performed and yields relatively few complications^3^.

**Table 4:** Day 14 post-op assessment of the trial groups.

Assessment	with (n=10)		without (n=10)		Total (n=20)		p value(1)
	N°	%	N°	%	N°	%	
Healing							0,085
Good	08	80,0	04	40,0	12	60,0	
Moderate	02	20,0	06	60,0	08	40,0	

Crusts							0,675

None	06	60,0	06	60,0	12	60,0	
Moderate	04	40,0	04	40,0	08	40,0	

Nasal permeability							0,185(2)

Poor	-	-	02	20,0	02	10,0	
Moderate	05	50,0	06	60,0	11	55,0	
Good	05	50,0	02	20,0	07	35,0	

(1) Fisher's Exact.

(2) Chi-squared.

**Table 5:** Day 30 post-op assessment of the trial groups.

ASSESSMENT	with (n=10)		without (n=10)		Total (n=20)		p value(1)
	N°	%	N°	%	N°	%	
Healing							0,763
Good	09	90,0	09	90,0	18	90,0	
Moderate	01	10,0	01	10,0	02	10,0	

Crusts							0,500

None	02	20,0	03	30,0	05	25,0	
Moderate	08	80,0	07	70,0	15	75,0	

Nasal permeability							0,062 (2)

Poor	-	-	02	20,0	02	10,0	
Moderate	02	20,0	05	50,0	07	35,0	
Good	08	80,0	03	30,0	11	55,0	

(1) Fisher's Exact.

(2) Chi-squared.

Attachment **Patient assessment protocol after submucosal electrocautery ablation with and without lateral fracture of the inferior turbinate.**

Name:

Age:

Gender:

1. Assessment on the 1st day post-op:

a. nasal bleeding:

[ ]none [ ]mild [ ]moderate [ ]severe.

b. pain:

[ ]none [ ]mild [ ]moderate [ ]severe.

2. Assessment on the 7th day post-op:

a. healing (macro view by anterior rhinoscopy), evaluating edema, hyperemia and seropurulent secretion:

[ ]good [ ]moderate [ ]poor.

b. crust formation in the nasal cavities:

[ ]none [ ]moderate [ ]severe.

c. nasal permeability:

[ ]poor [ ]moderate [ ]good.

3. Assessment on the 14th day post-op:

a. healing (macro view by anterior rhinoscopy), evaluating edema, hyperemia and seropurulent secretion:

[ ]good [ ]moderate [ ]poor.

b. crust formation in the nasal cavities:

[ ]none [ ]moderate [ ]severe.

c. nasal permeability:

[ ]poor [ ]moderate [ ]good.

4. Assessment after 30 days of surgery:

a. healing (macro view by anterior rhinoscopy), evaluating edema, hyperemia and seropurulent secretion:

[ ]good [ ]moderate [ ]poor.

b. crust formation in the nasal cavities:

[ ]none [ ]moderate [ ]severe.

c. nasal permeability:

[ ]poor [ ]moderate [ ]good.

Another method described in the literature is the lateral fracture of the inferior turbinate. This method was first introduced in 1904, by Killian, against the adverse effects of turbinectomies^2^. In this case, the turbinate suffers a medial fracture and is repositioned laterally with the long nasal speculum. It is a simple procedure that does not cause major complications or bear great risks^5^. However, it is not a very effective procedure when performed alone, not combined to other techniques^1^. Thus, lateral fracture is carried out during turbinectomy, submucosal electrocautery ablation, and septoplasty, to name a few. In agreement with this author, after 30 days we found good nasal permeability in 80% of the patients who underwent submucosal electrocautery ablation coupled with lateral fracture. On the other hand, in the patients who underwent only electrocautery ablation without lateral fracture, we found good nasal permeability in 30% and moderate permeability in 50% (p=0.062) (threshold likelihood indicating a trend). Such findings show that submucosal electrocautery ablation coupled with inferior turbinate lateral fracture is more efficient than electrocautery ablation alone.

In relation to mucosal healing where edema, hyperemia and seropurulent secretion were present, some differences between the groups were noticed. On Day 7 post-op the fractured group had good healing (60%) and the group without fracture showed moderate healing (70%) (p=0.041). On Day 14, the fractured group presented good healing (80%) and the group without fracture showed moderate healing (60%) (p=0.085). Day 30, all patients presented satisfactory healing. Jackson et al. observed long standing mucosal edema and hyperemia on patients in whom the ablation reached the turbinate bone1. In our study a better healing was noticed on the group that underwent lateral fracture of the turbinate. We can not ascertain the real cause of such difference. One might speculate that the group that did not suffer the lateral fracture may have had more ablated tissue, thus leading to less efficient healing.

## CONCLUSIONS

From this comparative analysis of patients submitted to submucosal electrocautery ablation with and without lateral fracture of the inferior turbinate, after 30 days of follow-up, we concluded that the lateral fracture has yielded better nasal permeability when compared to submucosal electrocautery ablation alone. We must stress that the patients who did not undergo lateral fracture of the turbinate had unsatisfactory healing in the first two weeks, and this fact requires further clarification.
